# A Fuzzy-Based Relay Security Algorithm for Wireless Sensor Networks

**DOI:** 10.3390/s25144422

**Published:** 2025-07-16

**Authors:** Nan-I Wu, Tung-Huang Feng, Min-Shiang Hwang

**Affiliations:** 1 Department of Information Management, Lunghwa University of Science and Technology, Taoyuan 33306, Taiwan; naiwu@gm.lhu.edu.tw; 2 Department of Computer Science and Information Engineering, Asia University, Taichung 41354, Taiwan; fth@gm.youth.tc.edu.tw; 3 Department of Medical Research, China Medical University Hospital, China Medical University, Taichung 40402, Taiwan

**Keywords:** fuzzy relationship rule, relay selection algorithm, tabu algorithm, wireless sensor network

## Abstract

Wireless sensor network data is an important source of big data. A sensor node cooperatively transmits or forwards data through intermediate nodes to a collection center, which is then aggregated for big data analysis and application. The relay selection algorithm selects the best transmissible node among the candidate nodes to fully exploit the limited resources of the sense nodes and extend the network lifecycle. A wireless sensor network relay selection algorithm based on a fuzzy inference system often uses sorting methods or random methods as the selection mechanism to choose when the fuzzy system outputs the same result. However, in the state of communication, networks often face the retransmission of lost packets, which consumes excess electricity. This study proposes a contraindicated safety selection mechanism algorithm to address equal output values in fuzzy systems. The proposed algorithm effectively reduces the retransmission probability to achieve benefits that isolate destructive or malicious nodes, thereby maintaining a higher network lifespan and safety.

## 1. Introduction

A wireless sensor network is a typical networking architecture of the Internet of Things [1,2]. It is also the source of a lot of big data information. When the system of wireless sensor networks is connected to the sensing nodes of sensors in various application environments [3,4], the perceived information reaches a collection center by transmitting data throughout a convenient and universal network infrastructure. After this massive data is aggregated, it becomes beneficial information [5]. Therefore, a wireless sensor network can be closely related to the Internet of Things. The selection of the most suitable relay node information as a transmissible partner among various neighborhood nodes can be solved by the relay selection algorithm [6]. An efficient algorithm design would obtain benefits in energy management and extend the network lifecycle [7,8,9]. In detail, when the sensor is deployed in an unattended environment, the management of a specific size and limited capacity will not allow all possible nodes to transmit and receive the same information. Repeatedly consuming others’ electricity will reduce the overall lifespan of the network.

The research highlights that the most significant challenge in studies is designing a relay selection algorithm that balances the power of nodes and communication quality while maintaining an optimal network lifecycle [10,11]. In recent years, the scholars Yang et al. [12] and Brante et al. [13] have applied fuzzy theory to relay selection algorithms in wireless sensor networks. They confirmed that constructing a relay selection algorithm based on a fuzzy inference system is more effective than traditional algorithms. Because the different design concepts of fuzzy systems are all related to fuzzification, the fuzzy rule base, and defuzzification, they will have varying degrees of relative problems in facing the same candidate’s value. To solve this problem, Yang employed a sorting-selection method from traditional transitions. At the same time, Brante utilized a random selection method in the structure and integrity of his fuzzy relay selection algorithm. When a wireless sensor network is installed in an unstable environment or subjected to malicious node attacks [14,15,16,17], they did not emphasize facing the same output value; therefore, those selection mechanisms had a cumulative long-term impact on overall effectiveness.

The research team used the principles of Tabu algorithms to design a new selection mechanism for a fuzzy relay algorithm [18]. A Tabu algorithm with memory optimization, proposed by Glover in 1986, can utilize the elastic approach of memory from previous record movements to avoid falling backwards or circumstances that would lead to a cycle [18]. The solution can obtain a global optimal solution by effectively escaping the local optimal solution to form a strategy for solving combinatorial optimization problems. This study will utilize the Tabu algorithm to design a safety-selection mechanism for a fuzzy relay algorithm, leveraging the Tabu algorithm’s characteristics of short-term memory to develop suitable optimization options within limited operation and memory space in wireless sensor networks. In the effectiveness analysis, the probability of obtaining the same output value is analyzed theoretically, which is compared with existing sorting methods and random methods in terms of the impact caused by the network lifecycle, allowing the mechanism to be effectively utilized in actual situations. In this article, we will propose an algorithm that has good security and power consumption.

Other sections of this paper are organized as follows: Section 2 provides related works, describing the existing selection mechanism of the fuzzy relay algorithm. Section 3 proposes the research methods, depicting the Tabu algorithm as a selection mechanism for a fuzzy relay selection algorithm. Section 4 presents an effectiveness analysis, explaining the fuzzy inference system from the perspective of achieving the same output value, and discussing the impact of the selection mechanism for retransmitting excess consumption. It then analyzes the effectiveness of the methods and the differences in applying existing algorithms using sorting and random mechanisms. Finally, a conclusion is proposed about the contribution of the research results.

## 2. Related Works

This section describes the fuzzy system architecture on wireless sensor networks and explains how Yang and Brante use the selection mechanisms when the fuzzy system outputs the same value.

### 2.1. Fuzzy-Based Relay Selection Algorithm

To establish a fuzzy system relay selection algorithm on wireless sensor networks [19,20,21,22], five steps are required, as follows:

**Step 1**: Defining the input and output variables and giving the domain of membership functions.
**Step 2**: Defining the membership functions as the fuzzy processing program, and then outputting the input value with a different membership degree [0, 1].
**Step 3**: Designing a fuzzy rules base. Based on experience or expert knowledge, designers use the semantic transform of “IF X1 is A AND X2 is B, THEN Y is C” to create a fuzzy rule base that will become the core of the fuzzy inference engine.
**Step 4**: Fuzzification inference: The results are derived from an interactive fuzzy operation control rule base.
**Step 5**: Defuzzification: It achieved an output value from aggregated results using the membership degrees.

**Figure 1:**
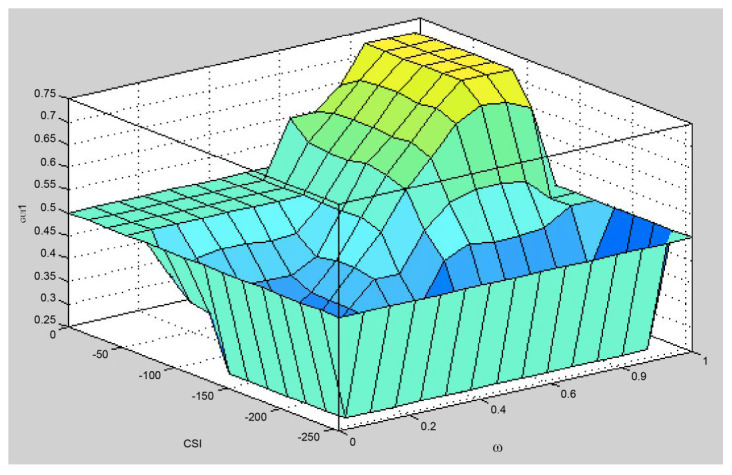
The surface chart of the AND connection fuzzy inference.

**Figure 2:**
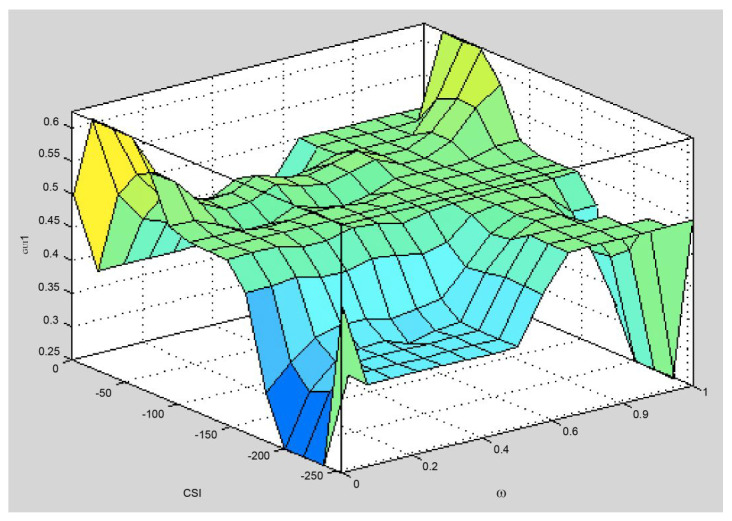
The surface chart of the OR connection fuzzy inference.

Finally, the fuzzy relay selection algorithm selects the node with the maximum defuzzification value as the relay node for this round. It is described above, with a fuzzification and fuzzy rule based on the fuzzy system. However, due to the different building steps, it will output different creature results from the traditional linear calculation [7]. In the same fuzzy system architecture, Figure 1 shows the surface generated using an AND connection of fuzzy inference, and Figure 2 shows the surface generated using an OR connection of fuzzy inference. On the same platform, the surface represents different input values that produce the same output value. In other words, the fuzzy relay selection algorithm must make appropriate choices now. It confirms that candidate nodes can reduce the burden and risks of retransmission.

The fuzzy-based relay selection algorithm builds upon the general framework described in [19,20,21,22] to ensure consistency with established designs of fuzzy inference systems. Our main improvement lies not in modifying the fuzzy inference process itself but in proposing a novel Tabu-based safety-selection mechanism that enhances decision-making when output values are equal—an issue often overlooked in prior works.

### 2.2. Yang’s Fuzzy Inference Method

Yang first proposed relay selection algorithms on wireless sensor networks [12]. Although Yang assigned membership functions used in fuzzy systems, he only used a traditional method of the weighted average to replace the fuzzy inference process of fuzzification and defuzzification. It can be said that only embryonic research is taking place regarding fuzzy inference, so it can not be called a complete architecture of fuzzy relay selection algorithms [7]. Yang applied the fuzzy theory as follows:

**Step 1**: Establishing membership degree matrix of input variables (Re: Remaining power; CSI: Communication State Information). Formula (1) is an example of a five-input matrix of candidate nodes:
  (1) $$R\left[\begin{array}{c} Re\\ CSI \end{array}\right]=\left[\begin{array}{ccccc} 0.3 & 0.7 & 1 & 0.6 & 0.9\\ 0.4 & 0.6 & 0.5 & 1 & 0.8 \end{array}\right]$$
**Step 2**: Formula (2) assigns the output weights for adjustment.
  (2) $$\begin{array}{c} W=(0.5,0.5) \end{array}$$
**Step 3**: Use the operation of M(·,+) to calculate the output result by B=W·R in Formula (3). The output node of 0.85 is determined as a relay node. Yang used a sorting algorithm as its selection mechanism when the result has the same output value.
  (3) $$\begin{array}{c} B=W\cdot R=(0.35,0.65,0.75,0.8,0.85) \end{array}$$

Simulations have demonstrated the effectiveness of Yang’s relay selection algorithms. Compared to the relay selection algorithm based solely on CSI network quality, it achieves better performance throughout the network’s lifecycle. Although Yang’s method is challenging to differentiate from traditional relay selection algorithms, the literature on relay selection algorithms does not have a separate category for fuzzy relay selection algorithms. Brante, as judged by Yang’s research, was the first to study fuzzy relay selection algorithms. Regarding the probability of the same output, it is $1/(n^{2}+n)$. It will output the same value only when Formula (4) is true, as the two inputs have the same weights.

(4) $$\begin{array}{c} \left\{\begin{array}{c} Re_{n}+CSI_{n}=Re_{m}+CSI_{m}\\ n,m\in \mathrm{Candidate}\;\mathrm{nodes},\;\mathrm{and}\;n<>m \end{array}\right. \end{array}$$

### 2.3. Brante’s Fuzzy Inference Method

Brante proposed a new fuzzy relay selection algorithm [7]. It utilized fuzzy systems to control cooperating nodes, which is in contrast to Yang’s method from 2009. The simulation yields a better result when compared with traditional algorithms, such as ’relay random selection’ and ’relay opportunity selection’, using the Brante method. Brante fuzzy systems utilize a straightforward calculation mechanism to prolong the network’s lifecycle.

**Figure 3:**
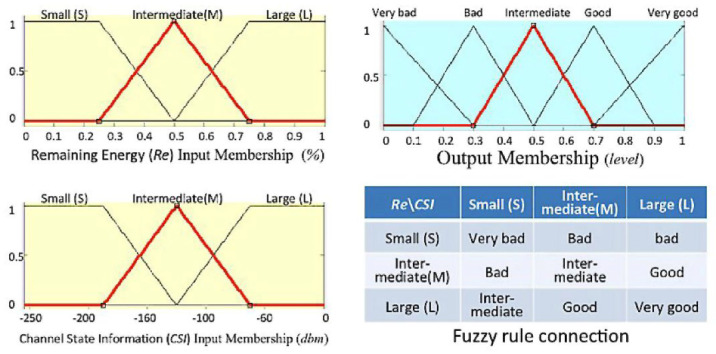
Brante’s fuzzy system block diagram for relay selection.

Figure 3 is Brante’s fuzzy relay selection algorithm, which utilizes a specific membership function and rule base. In order to adjust the remaining power and communication quality, two input variables, Re and CSI, are defined. The input parameters use two trapezoids and one triangle of a fragmentary continuous membership function, and the output parameters use five triangles of a fragmentary continuous membership function. The system defines nine fuzzy rule bases that all utilize the AND-MIN connection methods.

**Figure 4:**
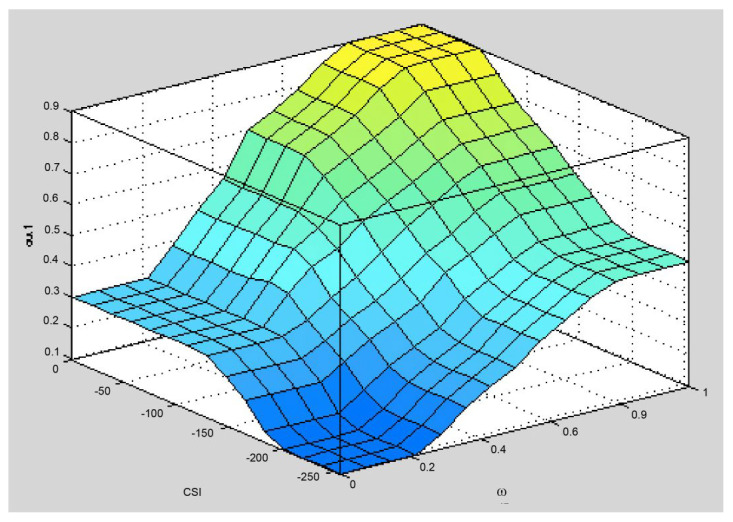
Surface chart of Brante’s fuzzy system.

Figure 4 is a surface plot of Brante’s fuzzy system. The system has four high platforms. The probability that they will output the same value is 2.5%. This is a lot higher than the Yang method. It can be said that it has reached the level of needing attention. Therefore, when the defuzzification results are the same, the Brante algorithm, which uses a random method as its selection mechanism, is employed to determine a relay node.

## 3. Research Methods

The structure of Section 3 follows a logical buildup: Section 3.1 and Section 3.2 provide necessary background immediately before presenting our main contribution in Section 3.3.

### 3.1. Relay Selection Algorithm in a Fuzzy System

**Figure 5:**
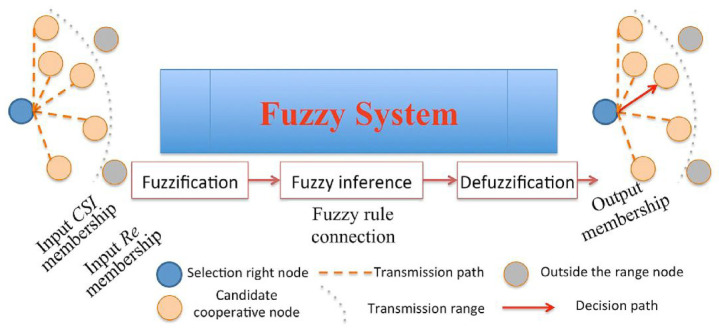
Relay selection algorithm in a fuzzy system.

The block diagram of a fuzzy system is used for relay selection on a wireless sensor network, as shown in Figure 5. There are two input variables (Re: Remaining power; CSI: Communication State Information) and an output, the membership result. To select the correct node within the transmission range of all candidate nodes, the selection process must determine the most appropriate relay node cooperation to regulate the remaining power and ensure the overall quality of communication networks. With the fuzzification in a fuzzy system, fuzzy-rule-based inference is derived, and finally, the node with the maximum defuzzification value is selected as the relay node to transmit.

This study applies the Tabu algorithm to fuzzy inference system processing, aiming to achieve the same output value and thereby improve the selection mechanism’s performance; this reduces unnecessary retransmissions of electricity consumption that can cause instability or an attack from malicious nodes in wireless sensor networks.

### 3.2. Tabu Algorithm

The proposed method is based on the Tabu search algorithm. The Tabu search (TS) algorithm was proposed as a type of heuristic algorithm (Heuristic Search) by Glover in [18], which differs from a traditional gradient method; it terminates early when there is no better solution than the current situation. However, it will utilize an elastic memory calculation process to establish or update the Tabu list and then escape from the local optimal solution to find the global optimal solution [18] effectively. The Tabu method has five components: the moving step, candidate list, Tabu list, opening Tabu list, and end principle. It follows these calculation steps:

**Step 1**: Enter the values of the parameters and conditions.
**Step 2**: Randomly select any one group of the starting solutions.
**Step 3**: Construct a candidate list for selecting the best solution from the neighborhood.
**Step 4**: Before performing Step 5, first examine the Tabu list to filter the selected moves; only allow moves that are not in the Tabu list or are in compliance with the principle of opening the Tabu list. If not in line with the principle of opening the Tabu list, return to Step 3 and select sub-optimal solutions to view.
**Step 5**: Be moving.
**Step 6**: To record moving steps to the Tabu list, and according to the first in, first out (FIFO) method, the oldest records are removed in order to maintain the length of the Tabu list.
**Step 7**: Calculate the objective function and compare the current best solution and the recorded optimal solution. If the current optimal solution is superior, go to Step 3; otherwise, go to Step 9. The score of the objective function is returned to the Tabu search algorithm to determine whether to update the best solution or perform a new move. The objective of its design is to minimize network damage, such as energy consumption.
**Step 8**: Update the best solution to the current recorded optimal solution.
**Step 9**: Determine the termination conditions for default; if it meets these conditions, go to the end step. On the contrary, return to Step 3.

This Tabu algorithm architecture can be divided into long-term memory and short-term memory. It depends on the memory range. The long-term memory architecture utilizes an entire record, which is unsuitable for a wireless sensor network, considering the limited resources of sensor nodes. Therefore, this study referred to a simple Tabu method (simple Tabu search) that utilized recently judged information to record short-term memory architecture. A Tabu list node can isolate these packet loss nodes to avoid falling into repetition when uncooperative nodes occur, and with the FIFO (First In and First Out) method, it can remove Tabu list records to achieve the purpose of the algorithm, minimizing damage.

### 3.3. The Proposed FTRS Algorithm

The proposed FTRS (Fuzzy-Tabu Relay Safety) method for relay node selection in wireless sensor networks (WSNs) is combined:

1. Fuzzy logic for multi-criteria decision-making (evaluating residual energy, link quality, etc.);
2. Tabu search with a short-term memory (Tabu list) to handle cases where multiple nodes receive the same fuzzy score.

This subsection describes the proposed fuzzy relay safety-selection algorithm for wireless sensor networks in this study. It applied a Tabu algorithm to the fuzzy relay selection algorithm as a way for the selection mechanism to achieve safety benefits. Because the existing research on random or ordering selection mechanisms ignores overall effectiveness during long-term impact in facing the same output results, this study focuses on the fuzzy inference system processing that occurs at the same output value to improve selection mechanism performance and avoid unnecessary retransmissions on electricity consumption that cause instablility or attacks from malicious nodes on wireless sensor networks.

**Figure 6:**
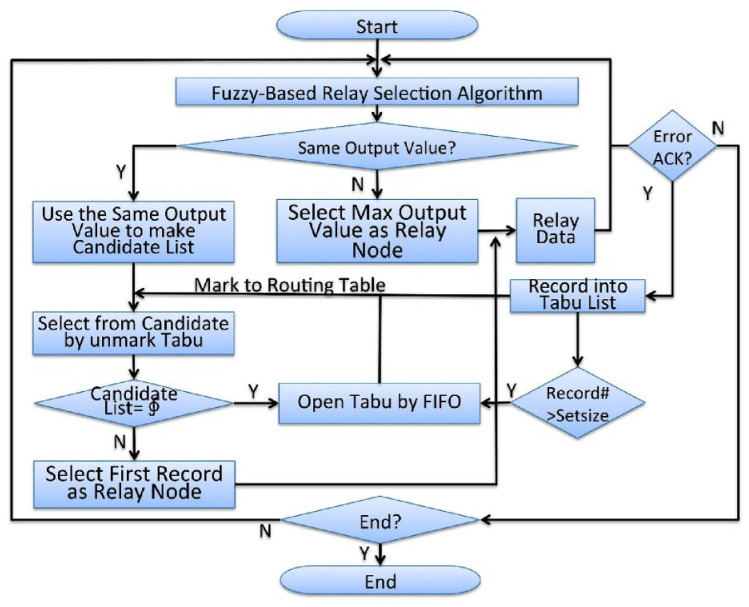
The flowchart of the fuzzy relay safety selection algorithm on wireless sensor networks.

Fuzzy relay safety-selection algorithms are considered in wireless sensor networks with limited computing and memory resources. It only fits a Tabu simple algorithm with short-term memory, which is then modified as the selection mechanism for a fuzzy relay selection algorithm. Its calculation process is shown in Figure 6. Those relevant procedures are described as follows:

**Procedure 1**: The fuzzy relay safety-selection algorithm is used to judge whether it generates using the case of the same output value first. If not, the first output node will be transferred directly as a relay partner.
**Procedure 2**: If it has the same output value, it is filtered from the routing table for a no Tabu flag record as the candidate node list. The Tabu flag is marked on a routing table when it is recorded in the transmissible failed Tabu list.
**Procedure 3**: If the candidate list is not empty, it uses the first record for the relay node to transmit the information directly. If the candidate list is empty, it starts the opening Tabu list and clears the Tabu flag in the routing table using the first-in, first-out method. Another timing procedure is used to start the opening Tabu list when the number of Tabu lists exceeds a preset value, which avoids routing table lists, including all of the Tabu lists. Then, the algorithm will never escape the release of nodes as a relay node for choosing.

The algorithm limits the Tabu list to a preset size of records. It is similar to the architecture of a simple Tabu method (simple Tabu search), which only records recently judged information in short-term memory. The Tabu list synchronizes with flags in the routing table, allowing it to reduce operational costs compared to candidate and Tabu lists. Especially, the Tabu list is provided from the physical layer of the Acknowledge (ACK) and the retry functions. It achieves results even after the relay nodes fail, thereby preventing the Tabu list from falling into repetitions chosen from uncooperative nodes. Naturally, it can isolate the packet loss nodes, providing safety benefits. Therefore, the study is titled A Fuzzy-Based Relay Safety Algorithm for Wireless Sensor Networks.

## 4. Performance Analysis

As mentioned above, the fuzzy relay safety algorithm begins and receives input when it outputs an equal value. The output probability of an equal value was $1/(n^{2}+n)$ in Yang’s sorting method and 0.25n in Brante’s random method. It is clear that the design concept of a fuzzy relay algorithm differs significantly in consideration of the output probability of equal values. If the algorithm is verified by the simulation mode of its own high performance, it will not be very objective. Therefore, to verify the potential benefits, the algorithm analysis was conducted using theoretical data to examine power consumption and memory space.

### 4.1. Analysis of Electricity Consumption

Table 1 presents the power consumption mode of the 802.15.4 protocol in wireless sensor networks [23]. Thus, the power consumption increases when developing a more complex selection mechanism to implement the program instruction code and calculate the selection. It is very easy to obtain compensation for preventing duplication and retransmission. Simply put, it is worth the price of monitoring the entire sensing process when the selection mechanism needs to only avoid a retransmission failure. Thus, this indicates that the design of a safety-selection algorithm is required to achieve a high ROI (return on investment).

Table 2 illustrates the comparison among Yang’s sorting selection method, Brante’s random method, and the proposed algorithm with the instruction category of selection mechanisms after the output value is obtained using fuzzy systems.

Table 2 shows that the safety-selection mechanism in the study’s algorithm outperforms Yang’s sorting method and Brante’s random method. Its execution clock is less than four times the duration of the instruction. However, the time complexity is also maintained at O(nlogn). The safety-selection mechanism utilizes 67-cycle electronics (ECycle), which increases power consumption by approximately 40.2 μA. It is only 0.000773 to obtain 52 mA for one, as it transmits and receives its power consumption. Compared to the 52 mA consumed during Transmitter/Receiver electronics, this extra current is negligible (less than 1 part in 1000) but yields significant savings by reducing retransmissions. Although the fuzzy methods of Yang and Brante et al. only have 14- and 18-cycle electronics, respectively, their methods will break ties randomly. This can lead to repeatedly selecting failing or malicious nodes, causing frequent retransmissions and wasted energy. Our method checks for ties, filters out failed nodes using a Tabu list, and expires stale entries if needed. This ensures that the relay nodes chosen are less likely to fail, cutting down the number of retransmissions. Although our method adds cycles, it provides safety benefits in uncertain conditions. This shows that the safety-selection mechanism has good feasibility and preservation benefits.

### 4.2. Analysis of Memory Storage Space

Let *n* be the number of nodes in the neighborhood table, and let *m* be the number of fields. Table 3 presents the results on the maximum memory storage space estimated for the selection mechanisms of Yang’s sorting method, Brante’s random method, and this study. The safety-selection mechanism requires allocating over 2n spaces on the stack memory. Depending on the safety mechanism described for the algorithm, the routing table needs to include a 1-bit field to set the Tabu flag. According to the Zigbee network protocol, it allows 10 automatic routing devices (i.e., n=10) and 8 bits of field on two parameters. The algorithm used in this study’s calculation has increased the total 16-bit memory space on each sensor node. If applied to a memory size of 16 kB to 128 kB, which is typical for a T-Brands sensor chip, it is still within the system’s capacity. The research contribution focuses on the selection mechanism when fuzzy outputs are equal, rather than modifying routing protocols, such as Zigbee, or defining new fitness functions.

This is easily understood from the above simple theoretical analysis. In order to create a great selection mechanism, increased power consumption and a lot of memory space are required; this has led all researchers to become hesitant. However, this study proposes a safety mechanism for the selection algorithm. It verifies that the small investment has avoided retransmission consumption and has obtained disproportionate safety benefits. Overall, the safety-selection mechanism is genuinely worthy of being applied to relay selection algorithms, as it has extended the network’s efficiency and reduced the system’s power consumption.

The performance advantages are supported by theoretical comparisons of execution cycles and memory cost, which reflect the lightweight and practical nature of the proposed mechanism.

## 5. Conclusions

Problems arise regarding outputting equal values in wireless sensor networks; this is almost nonexistent in traditional algorithms. It is also ignored in the fuzzy relay selection algorithm. Therefore, considering existing research may increase power consumption and memory load, researchers deal with an ordering or random approach to direct this. The proposed FTRS algorithm combines the adaptability of fuzzy logic with the memory-driven optimization of Tabu search, providing a safer, more efficient, and practical solution for relay node selection in wireless sensor networks, particularly under ambiguous scoring conditions. This study measures sensor power consumption in various modes and utilizes the concept of the Tabu algorithm to propose a safety-selection mechanism. Relatively, avoiding unnecessary power consumption through retransmission has yielded benefits, making the algorithm both fundamental and practical. It can benefit from isolating uncooperative or malicious nodes to maintain a higher level of network safety and longevity.

## Figures and Tables

**Table 1:** Power consumption in the model.

Operational Unit	Power Consumption
Transmitter electronics ($E_{Tx\_elec}$)	25 mA
Receiver electronics ($E_{Rx\_elec}$)	27 mA
Cycle electronics ($E_{Cycle}$)	0.6 μA

**Table 2:** A comparison of the instruction category execution times.

			Yang’s	Brante’s	Safety
Differentiate			Sorting	Randomly	Selection
Instruction	Complexity	Cycles	Method [12]	Method [7]	Mechanism
Random	O(n)	4		1 × 4 = 4	
If … then	O(n)	3			4 × 4 = 16
Reading records	O(n)	5	1 × 5 = 5	1 × 5 = 5	4 × 4 = 16
Sorting records	O(n × log n)	5	1 × 5 = 5	1 × 5 = 5	1 × 5 = 5
Filtering records	O(n)	4			1 × 4 = 4
Deleted records	O(n)	4			1 × 4 = 4
Writing records	O(n)	4	1 × 4 = 4	1 × 4 = 4	4 × 4 = 16
Marked Records	O(n)	3			1 × 3 = 3
Removed marks	O(n)	3			1 × 3 = 3
**Total**			14	18	67

**Table 3:** A comparison of the memory storage space estimate.

	The Maximum	Yang Sorting	Brante Random	Safety Selection
Distinguish	Memory Space	Method [12]	Method [7]	Mechanism
Routing Table	*n* × *m*	*n* × *m*	*n* × *m*	*n* × (*m* + 1)
Candidate list	*n*	*n*	*n*	*n*
Tabu list	*n*			*n*
**Total**		*n* + (*n* × *m*)	*n* + (*n* × *m*)	3*n* + (*n* × *m*)

## Data Availability

No new data were created or analyzed in this study. Data sharing is not applicable to this article.

## References

[1] Tian F., Xue H., Fu G., Liu G. (2024). SDN-based Privacy Protection Model for IoT Node Awareness. Int. J. Netw. Secur..

[2] Zhang L. (2024). Support Verifiable IoT Perception Layer Information Search and Encryption Algorithms. Int. J. Netw. Secur..

[3] Luo R.C., Chen O. (2012). Mobile Sensor Node Deployment and Asynchronous Power Management for Wireless Sensor Networks. IEEE Trans. Ind. Electron..

[4] Zheng K., Fu J., Liu X. (2025). Relay Selection and Deployment for NOMA-Enabled Multi-AAV-Assisted WSN. IEEE Sens..

[5] Duan H. (2025). DV-Hop Localization in Wireless Sensor Networks Based on Hybrid Firefly Particle Swarm Optimization. Int. J. Netw. Secur..

[6] Shen Y. (2025). The Problem of Extreme Value Falling into Local Optimal Solution in Anomaly Detection in Wireless Sensor Networks. Int. J. Netw. Secur..

[7] Brante G., Kakitani M.T., Souza R.D. (2011). Energy efficiency analysis of some cooperative and non-cooperative transmission schemes in wireless sensor networks. IEEE Trans. Commun..

[8] Zhou Z., Zhou S., Cui J.H., Cui S. (2008). Energy-efficient cooperative communication based on power control and selective single-relay in wireless sensor networks. IEEE Trans. Wirel. Commun..

[9] Song Z. (2025). Intrusion Detection Method for Wireless Sensor Network Based on Residual Convolutional Neural Networks Under Cloud Computing Environment and Its Application in Management. Int. J. Netw. Secur..

[10] Feng T.H., Shih N.Y., Hwang M.S. (2018). Safety Relay Selection Algorithms Based On Fuzzy Relationship For Wireless Sensor Networks. J. Supercomput..

[11] Feng T.H., Shih N.Y., Hwang M.S. (2015). A Safety Review on Fuzzy-based Relay Selection in Wireless Sensor Networks. Int. J. Netw. Secur..

[12] Yang W., Cai Y., Xu Y. An Energy-Aware Relay Selection Algorithm Based On Fuzzy Comprehensive Evaluation. Proceedings of the International Conference of Networks Security, Wireless Communications and Trusted Computing.

[13] Brante G., Peron G.d.S., Souza R.D., Abrão T. (2013). Distributed Fuzzy Logic-Based Relay Selection Algorithm for Cooperative Wireless Sensor Networks. IEEE Sens..

[14] Mao L., Zhang Y. (2024). Secure Localization Method in WSN Based on Improved MCL Algorithm. Int. J. Netw. Secur..

[15] Lu Y.C., Hwang M.S. (2022). A Cryptographic Key Generation Scheme without a Trusted Third Party for Access Control in Multilevel Wireless Sensor Networks. Int. J. Netw. Secur..

[16] Li W.T., Feng T.H., Hwang M.S. (2014). Distributed Detecting Node Replication Attacks in Wireless Sensor Networks: A Survey. Int. J. Netw. Secur..

[17] Madhavi S., Praveen R., Jagatheswari S., Nivitha K. (2025). Hybrid ELECTRE and Bipolar Fuzzy PROMOTHEE-based Packet Dropping Malicious Node Mitigation Technique for Improving QoS in WSNs. Int. J. Commun. Syst..

[18] Glover F.E.A. (1995). Genetic Algorithms and Tabu Search hybrids for optimization. Comput. Ops. Res..

[19] Gao Y., Zhang F. (2023). Multi-Copy Relay Node Selection Strategy Based on Reinforcement Learning. Sensors.

[20] Yang P., Kuang W., Wang S. (2023). Relay Selection for Dual-Hop Cooperative Ambient Backscatter Communication Systems. Sensors.

[21] Liu J., Hao Z., Wang J., Zhang X. (2023). Abnormal Traffic Detection Scheme Based on RBF Fuzzy Neural Network and Attention Mechanism in Robot Environment. Int. J. Netw. Secur..

[22] Yuan Y., Gu Y., Zhang Z. (2023). Public Data Integrity Auditing Scheme Based on Fuzzy Identity for Cloud Storage System. Int. J. Netw. Secur..

[23] (2015). IEEE Standard for Low-Rate Wireless Personal Area Networks (WPANs).

